# Therapeutic Properties of Sarsaparilla

**Published:** 1859-02

**Authors:** 


*Therapeutic Properties of Sarsaparilla.*—Dr. A. M. Adam, in speaking of Prof. Böcker, of Bonn ("Medical Notes from the Continent," &c., in *Edinborough Med. Journ.*, Oct. 1858), states his most recent pharmacodynamic experiments," which, I believe, are as yet unpublished, have been made with sarsaparilla. He informed me that, after carefully performing ninety-eight experiments with this drug on healthy people, he found that, contrary to all our usually received opinions on the subject, it possesses neither diuretic nor diaphoretic properties. Another series of twenty-six experiments, on the persons of uncured syphilitic patients, gave exactly the same results. Böcker also satisfied himself that sarza does not increase the efficacy of the agents, such as iod. potass., etc., which are usually given along with it; and that the good results obtained by the administration of this salt, dissolved in the decoction of sarza, are in no degree attributable to any virtue in the solvent fluid. I told Dr. Böcker that I remembered hearing Professor Syme, many years ago, express his opinion on the utter uselessness of so expensive a drug as sarza, remarking, in his own quaint, forcible style, that he believed an "infusion of hay" would be just as good, and a vast deal cheaper. He seemed amused, and said that he entirely agreed with Syme; that infusion of sarza had no greater effect on the system than so much common tea; and that we must regard it merely as a pleasant, but very expensive, vehicle for the administration of other medicines."

[Our own clinical observations have led us to the same conclusions as have been arrived at by the learned professor of Bonn, as to the utter absence of any therapeutic properties in the sarsaparilla.]—*Ibid.*

---

*Results of an extended Inquiry into the Quantity of Carbonic Acid evolved from the Lungs under the Influence of various Agents.*—Dr. Edward Smith, in a communication to the Section of Physiology of the British Association for the Advancement of Science, stated that he had conducted a series of experiments extending over several months, and found, by his new instrument, that the quantity of carbonic acid expired varied most materially under the influence of different kinds of food, different states of the atmosphere, etc.

The paper went into an inquiry—first, as to the quantity of carbonic acid expired in twenty-four hours, with the variations hour by hour; second, the influence of season; and third, the influence of nearly all ordinary articles of food and of a few medicines. During the summer, respiration is always feble, as compared with the colder months of the year; and although the skin exercises most important functions, he found that it was not vicarious for the lungs in the expiration of carbonic acid; for while the lungs expired 600 grains, the skin threw off only six grains. The increase in the quantity of carbonic acid was greater and more enduring after eating oatmeal and rice than after partaking of arrowroot; whilst wheat produced the greatest quantity, though the increase was less enduring than with oatmeal and rice.

Tea, coffee, and cocoa were found to be respiratory exciters, and consequently increased the waste of the system; they could not be classed as food; but as tea induced perspiration, it was most valuable as a remedy against the action of heat. Tea caused the evolution of much more carbon than it supplied. Tea would also be useful in cases of drowning and interrupted pulsation. Brandy, sometimes administered in cases of drowning, had the very opposite effect to that desired, being a non-exciter of pulsation; whereas tea increased the action of the lungs and skin. If the object were to prevent the waste of the system, then alcohol might be useful, and tea would be improper; but if they wished to refresh themselves, tea should be taken.

The experiments made showed that those who were more susceptible of injurious influence by heat were the least able to bear any change of climate; and if this were borne in mind, it would be found of service to those who might contemplate going abroad—to the east or elswhere.—*British Med. Journal,* from *Amer. Journal Med. Sciences.*

